# Treatment of Upper Crossed Syndrome: A Narrative Systematic Review

**DOI:** 10.3390/healthcare11162328

**Published:** 2023-08-17

**Authors:** Min Cheol Chang, Yoo Jin Choo, Keeyong Hong, Mathieu Boudier-Revéret, Seoyon Yang

**Affiliations:** 1 Department of Physical Medicine and Rehabilitation, College of Medicine, Yeungnam University, Taegu, Gyeongsan 38436, Republic of Korea; wheel633@ynu.ac.kr (M.C.C.);; 2 Cheonho S Orthopedic Clinic, Seoul 06014, Republic of Korea; 3 Department of Physical Medicine and Rehabilitation, Centre Hospitalier de l’Université de Montréal, Montreal, QC H2X 0A9, Canada; mathieu.boudier-reveret@umontreal.ca; 4 Department of Rehabilitation Medicine, School of Medicine, Ewha Woman’s University Seoul Hospital, Seoul 07804, Republic of Korea

**Keywords:** pain, pain management, musculoskeletal pain, musculoskeletal diseases, postural balance, musculoskeletal manipulations

## Abstract

Background and Objectives: Upper crossed syndrome (UCS) is a common musculoskeletal condition that is characterized by tightness and weakness of the muscles of the neck, shoulders, and upper back. The aim of this current study is to summarize and provide an overview of the treatment in patients with UCS. Materials and Methods: A MEDLINE (PubMed), Cochrane library, Embase, Scopus, and Web of Science database search was conducted for English-language articles about upper crossed syndrome that were published until 19 January 2023. To identify potentially relevant articles, the following key search phrases were combined: “upper crossed syndrome”, “upper cross syndrome”, “diagnosis”, and “treatment”. A total of 233 articles were identified. After reading the titles and abstracts and assessing their eligibility based on the full-text articles, 11 articles were finally included in this review. The risk of bias (RoB) was assessed using RoB-2 and ROBINS-I for the randomized controlled trials (RCTs) and the non-randomized clinical trial (non-RCT), respectively. Results: Among eleven studies that investigated the effect of treatment programs for UCS, five studies compared the therapeutic effect of exercise programs with controls, whereas six compared different rehabilitative treatment strategies, such as the muscle energy technique, soft-tissue mobilization, and stretching exercises. In addition, regarding the study design, ten studies were RCTs and only one study was a prospective observational study. Conclusions: Treatment programs including various types of exercises and techniques to correct an abnormal posture and restore neuromuscular imbalances are effective for decreasing pain and improving neck disabilities and postural deviations in patients with UCS.

## 1. Introduction

Upper crossed syndrome (UCS) refers to the condition that is characterized by tightness and weakness of the neck, shoulders, and upper back that cross between the dorsal and the ventral sides of the body [1]. Although there are no clear diagnostic criteria for UCS, it is commonly defined as altered muscle activation and movement patterns of the head, neck, shoulders, and back muscles. In UCS, neck and chest muscles, such as the suboccipitalis, sternocleidomastoid (SCM), levator scapulae, pectoralis major and minor, scalenes, and upper trapezius (UT), are tightened or shortened, whereas muscles of the neck and posterior upper back, such as the deep neck flexors (DNFs), serratus anterior (SA), rhomboids, middle trapezius (MT), and lower trapezius (LT), are weakened, stretched, and restrained. The tightness of the suboccipitalis, levator scapulae, and UT on the dorsal side is crossed by the tightness of the pectoralis major and minor, SCM, and scalenes on the ventral side; the weakness of DNFs on the ventral side is crossed by the weakness of the SA, rhomboids, MT, and LT [1]. Opposite-group muscle imbalances in UCS bring postural disturbances, misalignments in the upper limbs, and atlanto-occipital, cervicothoracic, and glenohumeral joint dysfunction [2]. The muscular imbalances may also lead to various musculoskeletal symptoms, such as headaches, neck pain, chest pain, upper back pain, tingling in the upper arms, or a limited range of motion in the neck or shoulders [3,4].

Postural abnormalities associated with UCS include a forward head posture (FHP), cervical lordosis, thoracic hyperkyphosis, protracted and elevated shoulders (round shoulders), and scapular winging with increased internal rotation and abduction [1,5]. The alteration in scapula muscles in UCS affects the movement of the scapula, which acts as a bridge and provides mobility and stability to the shoulders and neck [6]. A proper posture is necessary for optimal functional performance in daily activities. The awkward positions put constant pressure on the joints, and degenerative changes in joints progress rapidly when exposed to sustained pressure, causing muscle imbalances and pain [7]. If a muscle imbalance continues and progresses, this may lead to altered movement patterns and joint damage, creating further dysfunction.

The main risk factor for developing UCS is maintaining an abnormal posture for a prolonged duration. Some daily activities that involve bad postures are often occupation-related. Repetitive tasks or continuous work for long hours can aggravate postural deviations. For example, patients with improper postures are more prone to musculoskeletal injuries after handling or lifting heavy objects than others with a proper posture [8]. Musculoskeletal pain, most commonly neck pain, may present as the main symptom of UCS. Musculoskeletal pain may increase in relation to standing continuously or improperly sitting for long hours [9], which may eventually affect productivity and workability. Therefore, a postural correction should be included for pain management in UCS.

Previous studies have designed various types of exercises and therapeutic approaches to correct an improper posture and reduce the pain caused by UCS. However, to the best of our knowledge, no review has been conducted to investigate various treatment methods for patients with UCS. The aim of this current study is to summarize and provide an overview of the treatments for patients with UCS.

## 2. Materials and Methods

This review was conducted in accordance with the PRISMA (preferred reporting items for systematic reviews and meta-analyses) statement. The review protocol was registered in PROSPERO (447391). A MEDLINE (PubMed), Cochrane library, Embase, Scopus, and Web of Science database search was conducted for relevant articles about upper crossed syndrome. English-language articles that were published until 1 June 2023 were included. The search terms (“upper crossed syndrome” OR “upper cross syndrome”) AND (“diagnosis” OR “treatment”) were used to identify potentially relevant articles.

**Figure 1:**
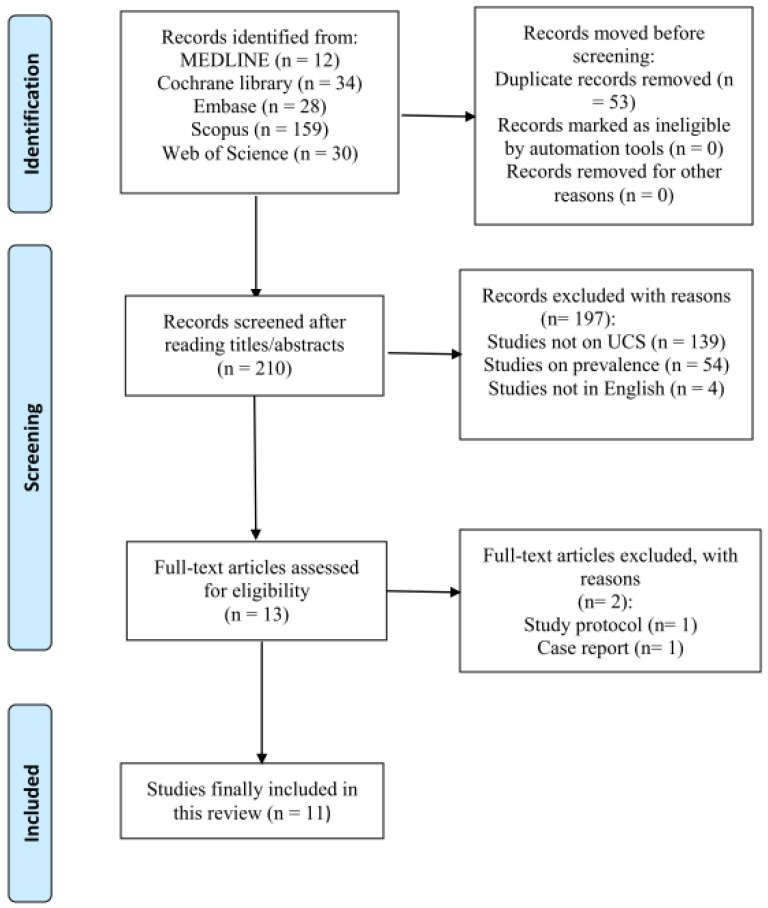
Search strategy.

A total of 263 potentially relevant articles were identified. After reading the titles and abstracts and assessing their eligibility based on the full-text articles, 11 articles were included in this review (Figure 1).

### 2.1. Study Selection and Data Extraction

The following inclusion criteria were applied for the selection of articles: (1) patients with UCS; (2) patients with neck pain; and (3) a comparison of the effect of therapeutic interventions with controls. The exclusion criteria were as follows: (1) studies that were not related to UCS; (2) reviews; (3) case reports; (4) commentaries; (5) letters; (6) animal studies; and (7) study outcomes that were not reported or insufficient. After reading the titles and abstracts, two independent reviewers (SYY and MCC) excluded articles and assessed the full-text articles to reject those ones not fulfilling the inclusion criteria. Any disagreement was resolved by consensus. The opinion of a third reviewer was put into consideration to resolve the disagreement if necessary.

All data were extracted independently by reviewers (SYY and MCC) using a standard data collection form. Discrepancies were resolved through consensus and discussions with another reviewer (KYH). The following data were collected from each eligible article: (1) the name of the first author, (2) the year of publication, (3) the study design, (4) the number of patients, (5) the duration of treatment, (6) the intervention programs, and (6) the outcome measures.

### 2.2. Quality Assessment

The methodological quality of randomized controlled trials (RCTs) was assessed using a revised tool for assessing the risk of bias in randomized trials (RoB 2) [10], while the non-RCT was evaluated using a tool for assessing the risk of bias in non-randomized studies of interventions (ROBINS-I) [11]. RoB 2 comprises a total of five evaluation domains: (1) bias arising from the randomization process, (2) bias due to deviations from the intended intervention, (3) bias due to missing outcome data, (4) bias in the measurement of the outcome, and (5) bias in the selection of the reported result. Depending on the degree of the risk of bias within each domain, the evaluations are categorized as “low”, “some concerns”, “high”, or, in cases where there is insufficient information for an assessment, “no information”. On the other hand, ROBINS-I consists of seven domains: (1) bias due to confounding, (2) bias due to the selection of participants, (3) bias in the classification of interventions, (4) bias due to deviations from the intended interventions, (5) bias due to missing data, (6) bias in the measurement of the outcomes, and (7) bias in the selection of the reported result. Each domain is rated as “low”, “moderate”, “serious”, “critical”, or “no information” depending on the level of bias risk. Either RoB 2 or ROBINS-I was used to assess the overall risk of bias for each included study. The overall risk of bias represents the least favorable evaluation across all bias domains.

## 3. Results

Among 11 studies, 5 studies compared the therapeutic effect of exercise programs with controls in patients with UCS [2,12,13,14,15]. Six studies compared different rehabilitative treatment strategies (e.g., the muscle energy technique (MET) vs. stretching exercises) [16,17,18,19,20,21]. Regarding the study design, ten studies were randomized controlled trials (RCTs) [2,12,13,14,15,16,17,18,20,21] and one study was a prospective observational study (POS) [19]. The characteristics of the included studies are summarized in Table 1.

### 3.1. Comparison between the Exercise Group and the Control Group

Five studies investigated the therapeutic effect of exercise in patients with UCS, and these were all RCTs [2,12,13,14,15].

In 2016, Bae et al. investigated whether stretching and strengthening exercises were helpful to patients with UCS [13]. Thirty students with UCS were enrolled in this study. Fifteen subjects in the exercise group performed exercise programs three times a week for 4 weeks (12 sessions), which consisted of self-stretching exercises of the UT and rhomboids and strengthening exercises of the MT and LT. To evaluate the treatment effects, infrared thermographic imaging, using a digital infrared thermographic imaging device, was used to measure the temperature of the posterior neck. The temperature differences of the pain sites and the contralateral sites correlated with pain severity in a previous study [22], so the authors believed that the changes in body temperature in painful or diseased areas may also correlate with a possible dysfunction. The results showed that there was a significant difference in the posterior neck temperature of the experimental group after the exercise program when compared to the control group. This study reported that stretching and strengthening exercises bring beneficial effects for patients with UCS by increasing body temperature, but it did not show the effect of the exercise program regarding pain severity, nor did it explain why the changes in body temperature are related to therapeutic effects in detail.

In 2019, Arshadi et al. conducted an RCT to investigate the effect of exercise [12]. They enrolled 30 men with UCS and randomly divided them into an exercise group (15 men) and a control group (15 men). The EMG activity was assessed before and after the exercise program. The exercise program consisted of stretching, strengthening, and stabilization exercises. It was performed in three sessions per week for 8 weeks, with each session lasting 50 min. Initially, the duration of the stretching exercises was 30 s, and five more seconds every two weeks was added. Stabilization exercises, focusing on the craniocervical joint and DNFs, were initially performed without any load in the supine and quadruped positions, and gradually increased in their repetition, duration, and range of motion (ROM). Strengthening exercises were targeted to restore the balance around the scapula. The results showed significant differences in the EMG activity of the UT, SCM, and SA, as opposed to the MT and LT activity, which revealed no changes. The training program was effective at decreasing the activity of the UT, while it increased the activity of the SA. After corrective exercises, the UT/SA and UT/LT ratios decreased. The decrease in the UT activity induced the increase in the LT activity. The decrease in the SCM activity reflected the increase in the DNF activity, which was observed after craniocervical flexion exercises that were targeted for the DNFs [23]. This study showed that an 8-week corrective exercise program was useful for managing muscular imbalances in patients with UCS.

The study by Karimian et al. also reported the effects of the National Academy of Sports Medicine exercises among teachers with UCS in 2019 [14]. Twelve teachers participated in the exercise program, which included self-myofascial release, stretching, and strengthening exercises. They showed a significant decrease in the forward head posture, shoulder angles, and hyperkyphosis after performing the exercises for 12 weeks, compared to 11 controls who did not participate in the program.

In 2020, Seidi et al. conducted an RCT to examine the effect of corrective exercises in 24 men with UCS [2]. The comprehensive corrective exercise program included initial-phase exercises (e.g., side-lying external rotation), improvement-phase exercises (e.g., standing diagonal flexions with a dumbbell and lying prone W exercises), and maintenance-phase exercises. The goal was to restore muscle balance and correct alignment during the movement pattern. The program was given three times a week for eight weeks to 12 patients with UCS, and the EMG activity was compared with controls who were not involved in the program. The study reported that the MT, LT, and SA activation levels showed significant differences after performing the corrective exercise program.

An RCT by Nitayarak et al., which was performed in 2021, reported that scapular stabilization exercises improved neck and shoulder postures, imbalances of the scapular muscles, and thoracic kyphosis [15]. Forty women were randomly allocated into an exercise group (20 patients) and a control group (20 patients). Scapular stabilization exercises targeting the MT, LT, rhomboids, and SA were applied using elastic bands in three sets of 10 repetitions on 3 days a week for 4 weeks. The cervical and shoulder angles were assessed from side-view photographs using the Kinovea computer program. A flexi ruler was used to measure the mid-thoracic curve, and a caliper was used to measure the length of the pectoralis minor muscle. Additionally, a handheld dynamometer was used to measure the strength of the scapular muscles. The results showed that the subjects in the exercise group showed a significant improvement in the cervical and shoulder angles, the length of the pectoralis minor, and the strength of the scapular muscles, although the degree of the mid-thoracic curve did not show any change compared to the control group.

### 3.2. Comparison of Different Rehabilitative Treatment Methods

Six studies investigated the effect of various rehabilitative treatment methods on UCS [16,17,18,19,20]. Five studies were RCTs [16,17,18,20,21] and one study was a POS [19].

In 2004, Yoo et al. recruited 20 visual display terminal workers to investigate the effect of a postural device on UCS [19]. This study aimed to assess the effect of a ball-back chair on the muscles associated with UCS. The ball-backrest chair used a ball with a size of 25 cm, which was placed at the horizontal height of T5–T7. The purpose of the ball-backrest chair was to provide a continuous external load against the upper trunk. The effect was assessed using the EMG activities of the UT, MT, and SA. The results showed that, compared to sitting in a chair with a general-purpose backrest, the EMG activities of the SA and MT increased and those of the UT decreased when sitting in a ball-backrest chair. Although this study did not involve any exercise programs, it suggested that a certain postural device or intervention can reduce the risk of developing musculoskeletal pain in patients with UCS.

In 2020, an RCT was conducted by Gillani et al. to compare the effect of an eccentric MET with static stretching exercises in 40 patients with UCS [16]. An eccentric MET with cervical segmental mobilization (20 patients) was equally effective at improving the cervical range of motion and reducing pain and neck disabilities as static stretching exercises with cervical segmental mobilization (20 patients). This study suggested that therapeutic approaches such as the MET and stretching exercises are both effective rehabilitative strategies for UCS management.

In 2021, Mahwood compared the effect of routine physical therapy with or without soft-tissue mobilization in 60 patients with UCS [17]. The routine physical therapy included hot packs and stretching exercises of: the pectoralis major and minor, the levator scapulae, the UT, the suboccipitalis, the rhomboids, the DNFs, the SCM, the scalenes, and the latissumus dorsi. Thirty patients who received soft-tissue mobilization with physical therapy showed a significant improvement in pain reduction and all neck ranges of motion compared to 30 patients who received only routine physical therapy. The study concluded that soft-tissue mobilization in combination with stretching exercises was effective at managing neck pain in UCS.

Most recently, in 2022, Aneis et al. conducted an RCT on 40 patients with UCS [21]. A multimodal approach, including postural correction training with ergonomic advice, the MET, cervical stabilization exercises, and scapulothoracic stabilization exercises, was given to 20 patients (intervention group), and the results were compared with 20 patients in the control group who received the MET only. The intervention group received the program three times a week for 3 weeks (total of 12 sessions). The intervention group showed significant improvements in their pain (measured by VAS), functional disability (measured by the neck disability index, NDI), craniovertebral angle (CVA), and sagittal shoulder angle (SSA) (measured by photogrammetry). Therefore, a multimodal approach was highly recommended in this study. The multimodal approach was more effective than the single rehabilitative approach.

In the same year, Sasun et al. compared the efficacy of myofascial rollers (40 patients) and post-isometric relaxation (40 patients) [18]. Dental professionals are often exposed to awkward chair positions and mechanical stress, making them prone to UCS. Therefore, this study included 80 dental undergraduate students with UCS. The results showed that myofascial rollers were more beneficial than post-isometric relaxation techniques, suggesting that applying myofascial rollers was an effective protocol for decreasing pain and improving postural deviations in UCS.

Yaghoubitajani et al. also conducted an RCT to compare the effects of online-supervised corrective exercises versus workplace corrective exercises among office workers with UCS in 2022 [20]. They allocated 36 patients to online-supervised (home-based), workplace-based, and control groups (12:12:12). Online-supervised and workplace corrective exercises, including those strengthening the DNFs and the scapula muscles, were performed three times a week for 8 weeks. The program consisted of a 5 min warm-up and cool-down, and with exercises addressing a correct posture, muscle activation, and movement patterns. The results showed that corrective exercises were beneficial for improving neck–shoulder pain, postural angles, upper trapezius activation, and workability in the online-supervised group compared to the control group. Both the online-supervised and workplace groups showed improvements in their FHP, round shoulders, round back, and neck–shoulder pain. The corrective exercises were effective at improving work performance and UT and SA activation only in the online-supervised group. The study recommended that supervised intervention was more beneficial than un-supervised intervention.

### 3.3. Risk of Bias

**Figure 2:**
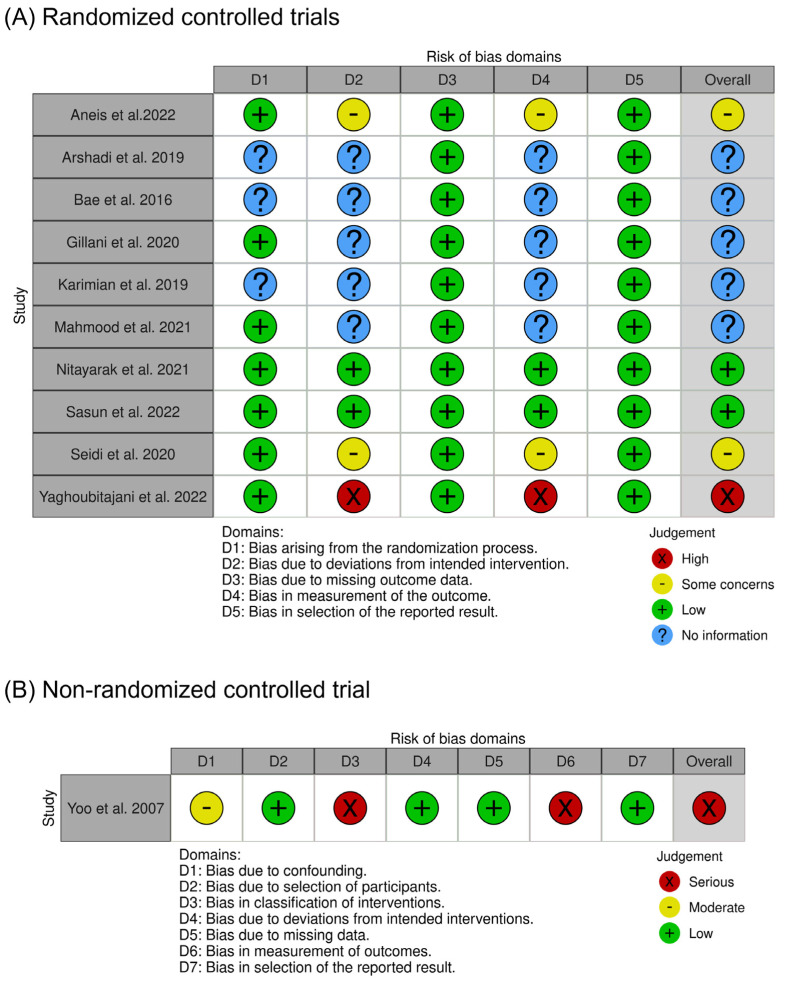
Methodological quality assessment for (**A**) randomized controlled trials [2,12,13,14,15,16,17,18,20,21] and (**B**) the non-randomized controlled trial [19].

Out of the RCTs assessed, two studies [15,18] had an overall low risk of bias, two studies [2,21] had some concerns regarding bias, and one study [20] had a high risk of bias. Additionally, five studies [12,13,14,16,17] had insufficient information for a proper evaluation of bias. The lack of information regarding the randomization process and the blinding of participants or assessors were the primary reasons for these results. Except for two RCTs [15,18], the blinding procedures for participants or assessors were not adequately carried out or mentioned (Figure 2A).

As for the non-RCT, the one study [19] did not provide information on whether the blinding of participants was implemented. Furthermore, there was a lack of a clear description regarding the allocation of participants to the intervention and control groups, leading to an overall judgment of a high risk of bias (Figure 2B).

In summary, while two RCTs demonstrated an overall low risk of bias, the majority of the remaining studies, both the RCTs and the non-RCT, had varying degrees of bias concerns or insufficient information about the blinding and allocation procedures, which can affect the reliability of their findings.

## 4. Discussion

Various rehabilitative strategies and techniques, such as stretching and strengthening exercises, stabilization exercises, postural correction exercises, the MET, and myofascial release, have proven to be effective for postural abnormalities in previous studies [21,24]. The most commonly used approach for postural abnormalities is the use of exercises to strengthen the weak muscles and stretch the tight muscles to correct misalignments. There is some controversy on the effect of stretching exercises, with claims that no changes were observed in the muscle length, but stretching exercises can improve the tolerance to stretching [25,26]. Most studies on the treatment of UCS also emphasized the importance of promoting and regaining the balance between asymmetrical muscles by stretching short muscles and strengthening weak muscles. The goal is to restore proper alignment as much as possible. One of the important risk factors associated with UCS is a prolonged faulty posture in the daily activities of life [27]. Muscles become tightened (shortened) or weakened (lengthened) as patients with UCS sustain an incorrect posture for long periods of time (Table 2). Thus, attempts to correct an improper posture are important. Measures to obtain a proper posture in daily life, including self-awareness and posture correction exercises, are needed to restore the normal posture of the head, neck, and shoulders to alleviate pain, an FHP, and round shoulders [28].

The results of the included studies showed that postural correction exercises and ergonomic interventions improved pain in patients with UCS [2,14,20,21]. This may be related to the mechanism whereby neck pain can be aggravated by prolonged sitting or an incorrect posture; an improper posture can increase the load on the cervical spine and induce muscle imbalances by changing the muscle length [29]. The repetitive mechanical stress on the neural, muscular, and articular tissues of the neck and shoulders can increase the tissue mechanosensitivity and negatively affect the tolerance of the central nervous system, resulting in pain and hyperalgesia [30]. Therefore, education about posture awareness, posture-corrective exercises, and strengthening and endurance exercises can be helpful for maintaining an upright neutral postural position [31]. Studies have reported that treatment programs for UCS were effective at restoring the normal sagittal configuration of the head, neck, and shoulders [2,14,15,20]. Normalizing the cervical spinal curve may also improve shoulder alignment because our body structures are all connected. Thus, sustaining a proper posture may result in an overall decrease in abnormal stresses on tissues, which can lead to a reduction in pain. Corrective exercises help patients to activate and increase the activity of inhibited, weak muscles and reduce the activity of overactive muscles to restore muscle balance and improve neuromuscular control.

Exercise programs, such as stretching exercises and cervical or scapulothoracic stabilization exercises, also improved neck disabilities [16,21]. An abnormal posture of the head and neck can decrease the proprioception of the neck [32]. The loss of proprioception feedback brings negative effects to the head and neck muscle function, reaction time, postural stability, and postural control [33]. Correcting an abnormal head posture seems to improve neck disabilities, as it enhances the proprioception of the neck.

Some specific muscles are targeted for the treatment of UCS. As mentioned earlier, UCS is a combination of an FHP, round shoulders, thoracic hyperkyphosis, protracted and elevated shoulders (round shoulders), and scapular winging with increased internal rotation and abduction. Cervical and thoracic spine misalignment can worsen the degree of the FHP, leading to round shoulders and increased thoracic kyphosis. DNFs are important muscles for maintaining normal cervical lordosis and correct cervical misalignment [34]. An FHP is characterized by weak, lengthened DNFs, which hinder head and cervical spine motor control [21]. This is the reason why strengthening exercises for DNFs are important for cervical stabilization. Exercise programs targeting DNF muscle activation increase the ability to keep an upright postural position [35]. Activating the DNFs via craniocervical flexion exercises promotes relearning of the muscle recruitment pattern [36] and suppression of the dominance of the SCM over the DNFs [37]. It may help to restore the mechanical balance between tight and weak muscles. In addition, the scapula muscles are important for the stability and mobility of the neck and shoulders [6]. Instability of the scapula and alterations in scapula muscle activation can cause mechanical dysfunction in the neck, which can result in the aggravation of neck pain [38]. Exercises also need to focus on restoring and balancing the function of the scapula muscles, such as strengthening exercises for the SA and LT, to improve the stability of the scapula and to maintain a proper scapular location [39]. Changing the length–tension relationship that is commonly observed in UCS [40] and applying corrective exercises to muscles that are lengthened and underactive can restore the scapulothoracic alignment and normalize an appropriate scapulothoracic rhythm [41].

Other therapeutic techniques, such as the MET, were applied for the treatment of UCS [16,21]. The MET is a mobilization technique used by manual therapists to manage neck pain and to restore the ROM [42]. The restricted joint is placed at the end of the limited ROM, and the patient voluntarily contracts the muscle or resists a movement in a controlled direction against the therapist’s applied counterforce [43]. The MET is an active technique that brings therapeutic benefits through lengthening and strengthening muscles, mobilizing restricted articulations, and improving pain and disabilities by involving the patient in contributing to the corrective force [42]. In combination with other treatment methods, the MET enhances the elasticity of contractile and non-contractile muscles and decreases the tension of tight and overactive muscles in patients with UCS [16,21].

This review has some limitations. First, the studies included in this review were performed on relatively small sample sizes and over small durations. Second, therapeutic programs were not blinded in the exercise and control groups, which is commonly observed with exercise interventions. Third, the patient population and therapeutic interventions were heterogeneous among studies. A meta-analysis could not be performed due to the heterogeneity of the included studies. Fourth, the diagnosis of UCS may have been variable among the studies, since there are no definite diagnostic criteria for UCS. In the future, it would be beneficial to address these issues. In addition, although abnormal postures and muscular dysfunction are known as the likely contributing factors to neck pain, their relevance is still controversial. Our review suggests that addressing postural issues and attempting to restore a normal muscular balance may help to improve neck pain and disabilities in patients with UCS.

## 5. Conclusions

In summary, current treatment programs for UCS aim to activate and strengthen weak muscles, such as the DNFs, MT, LT, and SA, and they also focus on reducing the activities of overactive muscles, such as a tight UT, SCM, and levator scapulae. Correcting postural muscle imbalances may decrease the mechanical stress on muscles, which can result in a reduction in pain, neck disabilities, and a restricted ROM, and corrective exercises can improve postural deviations in patients with UCS.

## Figures and Tables

**Table 1:** Characteristics of the included studies.

#	First Author	Year	Study Design	No. of Patients (Active/Control)	Duration of Intervention	Intervention Program	Outcome Parameters	Results
1	Yoo et al. [19]	2007	POS	20 (cross-over)	N/A	A ball backrest vs. general-purpose backrest	EMG activity	The EMG activity of muscles such as the SA and MT increased and that of the UT decreased when sitting in a ball-backrest chair compared to sitting in a chair with a general-purpose backrest.
2	Bae et al. [13]	2016	RCT	30 (exercise vs. control, 15:15)	4 weeks, 3 sessions/wk (total of 12 sessions)	Middle and lower trapezius strengthening, and levator scapulae and upper trapezius stretching exercises	Changes in body temperature (using a digital infrared thermographic imaging device)	The results showed that there was a significant difference in posterior neck temperature in the experimental group after the exercise program when compared to the control group.
3	Arshadi et al. [12]	2019	RCT	30 (exercise vs. control, 15:15)	8 weeks, 3 sessions/wk (50 min, total of 24 sessions)	Stretching, strengthening, and stabilization exercises	EMG activity	The baseline EMG activity of the SA increased while the UT and SCM activity decreased. In addition, the UT/SA and UT/LT ratios decreased. Eight-week corrective exercises can balance muscle activities and can be used to manage upper-quadrant musculoskeletal disorders in UCS.
4	Karimian et al. [14]	2019	RCT	23 teachers (exercise vs. control, 12:11)	12 weeks, 3 sessions/wk (45 min, total of 36 sessions)	Exercises (self-myofascial release, stretching, and strengthening) with an ergonomic training intervention	Head forward angle, kyphosis angle, and round shoulder angle using a UCS software (https://www.cisco.com/) application	Patients who performed exercises showed a significant decrease in forward head posture, shoulder angles, and hyperkyphosis. The exercises had positive effects on reducing the forward head angle, the rounded shoulder angle, and the kyphosis angle.
5	Seidi et al. [2]	2020	RCT	24 (exercise vs. control, 12:12)	8 weeks, 3 sessions/wk (1 h, total of 24 sessions)	Comprehensive corrective exercise program	EMG activity, scapular dyskinesis test, and head/shoulder/thoracic kyphosis angle	Corrective exercises for UCS were effective at improving misalignments, muscle activation imbalances, and movement patterns.
6	Gillani et al. [16]	2020	RCT	40 (eccentric MET vs. static stretching exercises)	3 weeks, 2 sessions/wk (total of 6 sessions)	Eccentric MET vs. static stretching exercises; both with cervical segmental mobilization, TENS, and IR	Tragus-to-wall distance, VAS, NDI, and cervical passive range of motion	Both groups showed significant improvements, but a comparison across groups showed non-significant results. Both techniques were equally effective for managing pain, the cervical range of motion, and disabilities.
7	Nitayarak et al. [15]	2021	RCT	40 (exercise vs. control, 20:20)	4 weeks, 3 sessions/wk (total of 12 sessions)	Scapular stabilization exercises	The cervical and shoulder angles (using the Kinovea program), the length of the pectoralis minor (caliper), the strength of the scapular stabilizer muscles (handheld dynamometer), and the degree of the mid-thoracic curve (flexi ruler)	The subjects in the exercise group showed a significant increase in the cervical and shoulder angles, the length of the pectoralis minor, and the strength of the scapular muscles, although the degree of the mid-thoracic curve did not show any change compared to the control group.
8	Mahmood et al. [17]	2021	RCT	60 (physical therapy with soft-tissue mobilization vs. physical therapy, 30:30)	4 weeks, 2 sessions/wk (total of 8 sessions)	Instrument-assisted soft-tissue mobilization (15–20 min) and routine physical therapy	The inclinometer and numeric pain rating scale (NRS)	Patients who received soft-tissue mobilization with physical therapy showed a significant improvement in pain reduction and neck range of motion compared to the controls. Soft-tissue mobilization in combination with stretching exercises was useful for managing neck pain in UCS.
9	Aneis et al. [21]	2022	RCT	40 (multimodal vs. MET only, 20:20)	4 weeks, 3 sessions/wk (total of 12 sessions)	Postural correction training, MET, cervical stabilization exercises, and scapulothoracic stabilization exercises	Photogrammetry (CVA and SSA), VAS, and NDI	A decrease in VAS and NDI and an increase in CVA were observed post-intervention. Only the multimodal group showed a significant change in SSA, and between-group differences favored the multimodal intervention.
10	Sasun et al. [18]	2022	RCT	80 (myofascial rollers vs. post-isometric relaxation, 40:40)	4 weeks, 4 sessions/wk (20 min, total of 16 sessions)	Myofascial rollers and hot packs vs. post-isometric relaxation and hot packs	Numerical pain rating scale (NRS) and a postural assessment	To improve pain and postural deviation, myofascial rollers were more effective than the post-isometric relaxation technique.
11	Yaghoubitajani et al. [20]	2022	RCT	36 (home vs. workplace vs. control, 12:12:12)	8 weeks, 3 sessions/wk (50–60 min, total of 12 sessions)	Online-supervised vs. workplace corrective exercises involving strengthening of cervical and scapular muscles	VAS, outcome evaluation questionnaire, postural angles using photogrammetry, workability index questionnaire, and surface EMG	The online-supervised group reported more improvements in neck–shoulder pain, postural angles, workability, and upper trapezius activation than the controls. Both intervention groups reported improvements in neck–shoulder pain, forward head posture, round shoulders, and round back.

Abbreviations: CVA, craniovertebral angle; IR, infrared; EMG, electromyography; NDI, neck disability index; NA, not applicable; UT, upper trapezius; MET, muscle energy technique; MT, middle trapezius; NRS, numerical pain rating scale; LT, lower trapezius; RCT, randomized controlled trial; POS, prospective observational study; SA, serratus anterior; SCM, sternocleidomastoid; SSA, sagittal shoulder angle; TENS, transcutaneous electrical nerve stimulation; VAS, visual analog scale.

**Table 2:** Muscles involved in upper crossed syndrome.

Hypertonic (Tight, Overactive) Muscles (in Order from Head to Shoulder)	Hypotonic (Weak, Inhibited) Muscles (in Order from Head to Shoulder)
Suboccipitalis	Deep neck flexors
Sternocleidomastoids	Middle and lower trapezius
Levator scapulae	Rhomboids
Upper trapezius	Serratus anterior
Scalenes	
Pectoralis major and minor	

## Data Availability

Data sharing is not applicable to this article, as no datasets were generated or analyzed in the current study.
